# Supplementary material for: Spotted fever Rickettsia and relapsing fever Borrelia in rodents from southern India
Source: Microbiol Spectr. 2026 Aug 10;14(9):e02072-26. doi: 10.1128/spectrum.02072-26 (PMC13532344; doi:10.1128/spectrum.02072-26)
Supplement: Supplementary file 1 [file spectrum.02072-26-s0001.docx]

**Supplementary Figures**

**Figure S1:** *Rickettsia, Borrelia, Leptospira* and *Bartonella* infection status among individual rodents. Bartonella infection status was collated from (1)

**Figure S2:** (A) Maximum-likelihood phylogenetic tree of *Leptospira* spp. based on 16S rRNA gene sequences, constructed using 10,000 bootstrap replicates under the GTR + G + I substitution model. (B) Genetic similarity of the *Leptospira* sequence derived from *Rattus satarae* in comparison with closely related *Leptospira* species.

**References**

1.	Ansil BR, Viswanathan A, Ramachandran V, Yeshwanth HM, Sanyal A, Ramakrishnan U. Host-pathogen-vector continuum in a changing landscape: Potential transmission pathways for Bartonella in a small mammal community. Ecol Evol. 2025 Apr;15(4):e71085.
