# Supplementary material for: Spotted fever Rickettsia and relapsing fever Borrelia in rodents from southern India
Source: Microbiol Spectr. 2026 Aug 10;14(9):e02072-26. doi: 10.1128/spectrum.02072-26 (PMC13532344; doi:10.1128/spectrum.02072-26)
Supplement: Supplementary file 2 [file spectrum.02072-26-s0002.docx]

**Supplementary Tables**

**Table S1.** Rodents sampled across habitat types within the Kadamane landscape and screened for *Rickettsia*, *Borrelia*, *Orientia*, *Leptospira*, and *Coxiella*.

| **Species** | **Forest** | **Grassland** | **Human habitation** | **Total** |
| --- | --- | --- | --- | --- |
| *Rattus rattus* | - | - | 10 | 10 |
| *Rattus satarae* | 66 | - | - | 66 |
| *Mus cf. fernandoni* | 2 | 20 | - | 22 |
| *Mus cf. famulus* | 2 | 19 | 5 | 26 |

**Table S2.** Primers and PCR conditions used for different bacterial detection. *Borrelia* and *Leptospira* screening followed a nested PCR approach. ‘*’ and ‘⁺’ indicate outer and inner primer sets of nested protocol, respectively.

| **Bacterial genera** | **Locus** | **Primer sequence (5’-3’)** | **Size (bp)** | **Annealing temperature** | **Reference** |
| --- | --- | --- | --- | --- | --- |
| *Rickettsia* | ompB | 120-2788F: AAACAATAATCAAGGTACTGT | 811 | 58°C | (1) |
|  |  | 120-3599R: TACTTCCGGTTACAGCAAAGT |  |  |  |
| *Borrelia* | 16S rRNA | 16S1AF: CTAACGCTGGCAGTGCGTCTTAAGC* | 720 | 63°C | (2) |
|  |  | 16S1BR: AGCGTCAGTCTTGACCCAGAAGTTC* |  |  |  |
|  |  | 16S2AF: AGTCAAACGGGATGTAGCAATAC⁺ | 650 | 61°C |  |
|  |  | 16S2BR: GGTATTCTTTCTGATATCAACAG⁺ |  |  |  |
| *Orientia* | 56-kDa  protein | OtsuF: AATTGCTAGTGCAATGTCTG | 371 | 55°C | (3) |
|  |  | OtsuR: GGCAT- TATAGTAGGCTGAG |  |  |  |
| *Leptosira* | 16S rRNA | LeptoAF: GGCGGCGCGTCTTAAACATG* | 330 | 63**°**C | (4) |
|  |  | LeptoBR: TTCCCCCCATTGAGCAAGATT* |  |  |  |
|  |  | LeptoCF: CAAGTCAAGCGGAGTAGCAA⁺ | 289 | 63**°**C |  |
|  |  | LeptoDR: CTTAACCTGCTGCCTCCCGTA⁺ |  |  |  |
| *Coxiella* | IS1111A | F: CGCAGCACGTCAAACCG | 146 | 60**°**C | (5) |
|  |  | R: TATCTTTAACAGCGCTTGAACGTC |  |  |  |

**Table S3:** Sample specific prevalence of *Rickettsia* spp, *Borrelia* spp, *Leptospira* spp and *Bartonella spp*. Samples tested for *Coxiella* spp (spleen and pooled tissue) and *Orientia* spp (blood and pooled tissue) were negative. Note that the *Bartonella* positivity data reported here were previously published in Ansil et al. 2025 (6).

|  |  | ***Rickettsia*** | | ***Borrelia*** | | ***Leptospira*** | | ***Bartonella*** | |
| --- | --- | --- | --- | --- | --- | --- | --- | --- | --- |
| **Species** | **Sample type** | **% (pos/n)** | **95% CIs** | **% (pos/n)** | **95% CIs** | **% (pos/n)** | **95% CIs** | **% (pos/n)** | **95% CIs** |
| *Rattus rattus* | Blood | 0 (0/10) | 0- 27.75 | 10 (1/10) | 0.51- 40.41 | - | - | 0 (0/10) | 0- 27.75 |
|  | Pooled tissue | 10 (1/10) | 0.51- 40.41 | 0 (0/10) | 0- 27.75 | 0 (0/10) | 0- 27.75 | - | - |
|  | Kidney | - | - | - | - | 0 (0/10) | 0- 27.75 | - | - |
| *Rattus satarae* | Blood | 0 (0/66) | 0- 5.50 | 6.06 (4/66) | 2.38- 14.57 | - | - | 75.76 (50/66) | 64.19- 84.49 |
|  | Pooled tissue | 12.12 (8/66) | 6.27- 22.13 | 0 (0/66) | 0- 5.50 | 1.51 (1/66) | 0.07- 8.09 | - | - |
|  | Kidney | - | - | - | - | 1.51 (1/66) | 0.07- 8.09 | - | - |
| *Mus cf. fernandoni* | Blood | 0 (0/22) | 0- 14.86 | 13.63 (3/22) | 4.74- 33.33 | - | - | 54.55 (12/22) | 34.66- 73.08 |
|  | Pooled tissue | 0 (0/22) | 0- 14.86 | 0 (0/22) | 0- 14.86 | 0 (0/22) | 0- 14.86 | - | - |
|  | Kidney | - | - | - | - | 0 (0/22) | 0- 14.86 | - | - |
| *Mus cf. famulus* | Blood | 0 (0/26) | 0- 12.87 | 0 (0/26) | 0- 12.87 | - | - | 3.85 (1/26) | 0.2- 18.89 |
|  | Pooled tissue | 0 (0/26) | 0- 12.87 | 0 (0/26) | 0- 12.87 | 0 (0/26) | 0- 12.87 | - | - |
|  | Kidney | - | - | - | - | 0 (0/26) | 0- 12.87 | - | - |
|  | **Total** | **7.26 (9/124)** | **3.86- 13.21** | **6.45 (8/124)** | **3.30- 12.21** | **0.80 (1/124)** | **0.04- 4.42** | **50.81 (63/124)** | **42.12- 59.45** |

**Table S4:** Set of generalized linear models (GLMs) used to evaluate the effects of species, sex, and their interaction on infection status for *Rickettsia* and *Borrelia*. Models were ranked using ΔAICc, with the number of parameters (*k*) and Akaike weights (*wi*) reported for each model.

| **Model** | *k* | ΔAICc | *wi* |
| --- | --- | --- | --- |
| Rickettsia infection status ~ species + sex | 5 | 0 | 0.967 |
| Rickettsia infection status ~ species * sex | 8 | 6.75 | 0.033 |
| Borrelia infection status ~ species + sex | 5 | 0 | 0.958 |
| Borrelia infection status ~ species * sex | 8 | 6.23 | 0.042 |

**References**

1.	Roux V, Raoult D. 2000. Phylogenetic analysis of members of the genus Rickettsia using the gene encoding the outer-membrane protein rOmpB (ompB). Int J Syst Evol Microbiol 50 Pt 4:1449–1455.

2.	Richter D, Schlee DB, Matuschka F-R. 2003. Relapsing fever-like spirochetes infecting European vector tick of Lyme disease agent. Emerg Infect Dis 9:697–701.

3.	Fournier P-E, Siritantikorn S, Rolain J-M, Suputtamongkol Y, Hoontrakul S, Charoenwat S, Losuwanaluk K, Parola P, Raoult D. 2008. Detection of new genotypes of Orientia tsutsugamushi infecting humans in Thailand. Clin Microbiol Infect 14:168–173.

4.	Mérien F, Amouriaux P, Perolat P, Baranton G, Saint Girons I. 1992. Polymerase chain reaction for detection of Leptospira spp. in clinical samples. J Clin Microbiol 30:2219–2224.

5.	Ramatla T, Khumalo ZTH, Matshotshi A, Lekota KE, Taioe MO, Thekisoe O. 2023. Molecular detection of Coxiella burnetii and Coxiella species in rats and chickens from poultry farms in North West Province, South Africa. Vet Med Sci 9:2185–2191.

6.	Ansil BR, Viswanathan A, Ramachandran V, Yeshwanth HM, Sanyal A, Ramakrishnan U. 2025. Host-pathogen-vector continuum in a changing landscape: Potential transmission pathways for Bartonella in a small mammal community. Ecol Evol 15:e71085.
